# Elevated Cellular Uptake of Succinimide- and Glucose-Modified Liposomes for Blood–Brain Barrier Transfer and Glioblastoma Therapy

**DOI:** 10.3390/biomedicines12092135

**Published:** 2024-09-20

**Authors:** Larissa J. Lubitz, Moritz P. Haffner, Harden Rieger, Gero Leneweit

**Affiliations:** 1 ABNOBA GmbH, 75223 Niefern-Öschelbronn, Germany; 2 Carl Gustav Carus-Institute,75223 Niefern-Öschelbronn, Germany; 3 Department of Chemical and Process Engineering, Institute of Mechanical Process Engineering and Mechanics, Karlsruhe Institute of Technology, 76131 Karlsruhe, Germany

**Keywords:** glucose, drug targeting, liposomes, blood–brain barrier, glioblastoma

## Abstract

The uptake of four liposomal formulations was tested with the murine endothelial cell line bEnd.3 and the human glioblastoma cell line U-87 MG. All formulations were composed of DPPC, cholesterol, 5 mol% of mPEG (2000 Da, conjugated to DSPE), and the dye DiD. Three of the formulations had an additional PEG chain (nominally 5000 Da, conjugated to DSPE) with either succinimide (NHS), glucose (PEG-bound at C-6), or 4-aminophenyl β-D-glucopyranoside (bound at C-1) as ligands at the distal end. Measuring the uptake kinetics at 1 h and 3 h for liposomal incubation concentrations of 100 µM, 500 µM, and 1000 µM, we calculated the liposomal uptake saturation *S* and the saturation half-time *t*_1/2_. We show that only succinimide has an elevated uptake in bEnd.3 cells, which makes it a very promising and so far largely unexplored candidate for BBB transfer and brain cancer therapies. Half-times are uniform at low concentrations but diversify for high concentrations for bEnd.3 cells. Contrary, U-87 MG cells show almost identical saturations for all three ligands, making a uniform uptake mechanism likely. Only mPEG liposomes stay at 60% of the saturation for ligand-coated liposomes. Half-times are diverse at low concentrations but unify at high concentrations for U-87 MG cells.

## 1. Introduction

A glioblastoma is the most common malignant brain tumor in adults, accounting for around 80% of all primary malignant tumors of the central nervous system [1]. The incidence is currently 3.26 cases in 100,000 people, with increasing numbers due to various aspects, such as an aging population, environmental pollution, and improved diagnostics [1,2]. Despite diagnostics with the most advanced pre-operative and intra-operative neuroimaging as well as surgical resection combined with chemotherapy and radiotherapy, the prognosis for patients is still poor [3,4]. The median survival rate ranges from 8 to 15 months [1,5], and the 5-year survival rate is currently at about 5% [1]. The development of treatments to cure or delay the progress of a glioblastoma is extremely aggravated compared to extracranial tumors due to various aspects: (a) the existence of the blood–brain barrier [6,7], (b) the unique tumor phenotype [8,9], and (c) the high aggressiveness of the tumor itself [10].

The blood–brain barrier (BBB) is the major obstacle and rate-limiting factor for drug transport into the brain [11]. Physiologically, it is more a biochemical barrier rather than a physical barrier [12], which protects the brain from toxins and pathogens and thus maintains the homeostasis of the intracranial microenvironment [7,13]. As a result, about 98% of all small molecules and 100% of all large macromolecules are retained at the BBB [13]. In addition to the molecular weight, the lipid solubility, charge, hydrogen bonds, and ionization profile are also decisive physicochemical characteristics for the transport of substances via the BBB. The BBB is formed by specific endothelial cells connected by tight junctions (TJs), which restrict the permeability and also the transport of potential therapeutics [7,13,14]. The transport of molecules across the blood–brain barrier can occur through a variety of unique pathways: the paracellular aqueous pathway, transcellular lipophilic pathway, adsorptive transcytosis, receptor-mediated transcytosis (RMT), and carrier-mediated transcytosis (CMT) [6].

Carrier-mediated transcytosis (CMT) is the most promising method to facilitate the transport of drugs into the brain due to the high transport affinity between the transporter and substance as well as the high transport capacity [15,16,17]. Along with sodium vitamin C transporter 2 (SVCT2), large neutral amino acid transporter 1 (LAT1), and monocarboxylic acid transporter 1 (MCT1), the glucose transporter 1 (GLUT1) is considered one of the most efficient transport systems at the BBB with a high affinity (*K*_m_ = 1–2 mM) for glucose [18,19,20]. GLUT1 is abundant at the BBB [21], with each brain capillary endothelial cell expressing around 6 × 10^6^ GLUT1 molecules [22]. Since glucose is required as a primary energy source in the brain and neurons themselves are unable to synthesize or store glucose, the transport of glucose across the BBB is vital [23]. The brain’s glucose consumption represents around 30% of the body’s total consumption.

In recent years, various glycoconjugates have been reported to increase the permeability of the BBB through GLUT1 transport, such as for dopamine [24,25], 7-chlorokynurenic acid [26,27], and ibuprofen [28]. Using structure–activity relationship studies, the highest affinity for GLUT1 was determined for analogs with substitution at the C-6 position of glucose [29]. However, the preparation of such prodrugs from macromolecules and also ionic drugs is not ideal, which is why nanocarriers are preferred with appropriate surface modifications. Besides CMT, the RMT offers advantageous properties and the interaction of functionalized nanocarriers with BBB targets, and further active internalization is not limited by size. As an example, the 5-HT_7_ receptor is reported to be expressed in the central nervous system (CNS) with high occurrence in brain cancers [30].

A large number of nano drug delivery systems (NDDSs), such as inorganic nanoparticles [31,32,33], phospholipid nanoparticles or liposomes [34,35], nanogels [36,37], and polymers [38,39,40], have been shown to overcome the BBB. Hu et al., 2009, showed that a particle size of less than 150 nm is to be favored for transport into the brain [41]. Among all those different NDDSs, liposomes should be given the highest attention due to their mature production methodology, high membrane permeability, and also simple methods for surface modification [42] with either endogenous molecules like glutathione [43], antibodies (e.g., anti-transferrin [44]), or cell-penetrating peptides [45,46]. Liposomes also offer other advantages, such as non-toxicity, biocompatibility, and biodegradability [47], and represent an effective non-invasive strategy to transport either hydrophilic, lipophilic, or amphiphilic drugs across the BBB by surface modification [11,48].

In recent years, a large number of developments and investigations of modified liposome-based platforms for enhanced cellular uptake and blood–brain barrier (BBB) transfer utilizing various targeting agents, such as peptides [46,49,50], antibodies [51], and cationic modifications [52], have shown several advancements.

In the present study, the glucose transporter 1 (GLUT1) and serotonin (5-HT) transporter systems are investigated as a target at the blood–brain barrier as well as in glioblastoma. For this purpose, the expression under different cultivation conditions was assessed in vitro concerning the medium’s glucose content and the cellular uptake of modified liposomes in both endothelial and glioblastoma cells. In a previous study [46], we were able to demonstrate that intracellular uptake of liposomes is enhanced by a cell-penetrating peptide with mPEG liposomes as a negative control, using both flow cytometry and confocal microscopy for the proof of intracellular uptake. The present study builds on the earlier results, now focusing on the liposomal surface modification with either glucose targeting the glucose (GLUT1) transporter system or succinimide targeting the serotonin (5-HT) receptor. The uptake kinetics is analyzed using exponential regressions to deduce the saturation limits and their half-times of liposomal uptake.

## 2. Materials and Methods

### 2.1. Preparation of Liposomes

**Figure 1:**
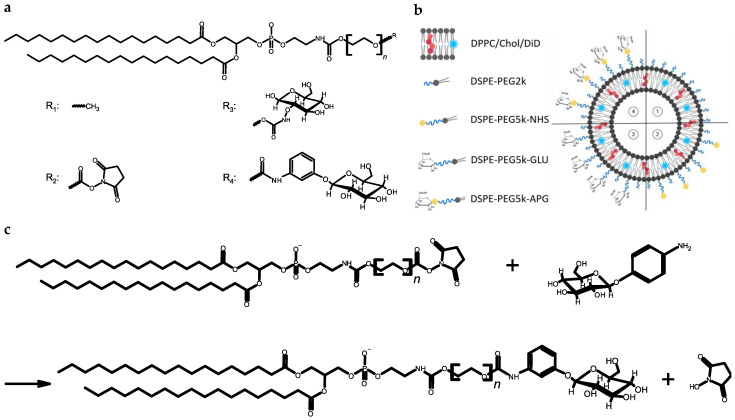
The schematic overview of structures, different liposomal formulations, and the reaction scheme of the conjugation: (**a**) displays the chemical structures of the liposomal components, (**b**) illustrates a schematic overview of the four liposomal formulations (1: mPEG, 2: NHS, 3: GLU and 4: APG) and (**c**) shows the conjugation reaction of the succinimidyl-modified phospholipid with the 4-aminophenyl β-D-glucopyranoside (APG).

1,2-Dipalmitoyl-sn-glycerol-3-phosphocholine (DPPC) and 1,2-Distearoyl-*sn*-glycero-3-phosphoethanolamine-*N*-[(polyethylene glycol)] (DSPE-PEG2k) were purchased from Lipoid GmbH (Ludwigshafen, Germany); 1,2-Distearoyl-sn-glycero-3-phosphoethanolamine-N-[(polyethylene glycol)] (DSPE-PEG5k-GLU) and 1,2-Distearoyl-sn-glycero-3-phosphoethanolamine-N-[succinimidyl (polyethylene glycol)] (DSPE-PEG5k-NHS) were purchased from Biopharma PEG Scientific (Watertown, MA, USA). Liposomes were prepared using thin-film hydration and with subsequent membrane extrusion. For this purpose, stock solutions of the components DPPC (Lipoid GmbH, Ludwigshafen, Germany), cholesterol, and DSPE-PEG5k-NHS were prepared in ethanol and rotated to produce dry lipid films in the intended compositions using a rotary evaporator. Rehydrating the lipid film to a final total lipid concentration of 20 mM was performed using 10 mM histidine buffer with 0.3 osmol/L sodium chloride at pH 7.4. The extrusion of liposomes was performed using track-etched polycarbonate membranes with different pore sizes (Whatman™, Cytiva, Marlborough, MA, USA) in two steps: (a) five-fold extrusion of liposomes through a membrane with a pore size of 400 nm and (b) 20-fold extrusion through a membrane with a pore size of 100 nm. The extrusion was performed using argon for pressurization in the pressure range from 10 bar to 25 bar. The phospholipid compounds are structurally shown in Figure 1a, while the liposomal composition is listed in Table 1 and shown in a schematic representation in Figure 1b.

### 2.2. Surface-Modification of Liposomes with Glucose

DSPE-PEG5k-NHS was included at 5 mol% in liposomes to enable conjugation of 4-aminophenyl β-D-glucopyranoside (APG, Santa Cruz Biotechnology, Dallas, TX, USA) to form an amide bond after formation of liposomes with DSPE-PEG5k-NHS, thus forming DSPE-PEG5k-APG, as shown in Figure 1c. This procedure will be termed “post-conjugation” in the following descriptions. The conjugation was carried out by incubating a 10 mg/mL solution of APG in buffer at a molar ratio of 1.2:1 with the DSPE-PEG5k-NHS-containing liposomes for 24 h at room temperature on the rotary wheel. After 24 h of conjugation, the crude liposomal product was dialyzed using Spectra/Por^®^ Biotech CE (Repligen, Waltham, MA, USA) tube with a molecular weight cut-off of 100 kDa to eliminate unreacted 4-aminophenyl β-D-glucopyranoside (APG). The dialysis medium consisted of the same buffer used for liposome preparation, and it was added outside of the dialysis membrane in a 300-fold excess of the sample and was exchanged after 2, 4, and 24 h. Finally, all liposomal formulations were sterile filtrated through a syringe filter with a pore size of 0.22 µm.

### 2.3. Characterization of Liposomes

After extrusion and final sterile filtration, the mean hydrodynamic diameter (Z-Average) and the polydispersity index (PdI) of the liposomes were determined using dynamic light scattering (DLS). For this purpose, the liposomal formulations were diluted 1:100 with buffer and measured using the ZetaSizer ZS90 (Malvern Instruments, Worcestershire, UK). Each sample measurement was performed in triplicate, each consisting of 5 single runs.

To determine physical liposomal stability, all liposomal samples were stored at 4 °C for four weeks and measured using DLS weekly. Additionally, the Zeta potential (ZP) of all four different formulations as shown in Figure 1b was determined using the ZetaSizer ZS90 (Malvern Instruments, Worcestershire, UK).

The glucose concentration of both glucose-containing liposomes (GLU and APG) was determined using a glucose hexokinase assay kit (Sigma Aldrich, St. Louis, MO, USA). The quantification is based on an enzymatic method whereby glucose is phosphorylated by adenosine triphosphate (ATP) in the first step, which is catalyzed by the hexokinase enzyme. The resulting glucose-6-phosphate (G6P) is then oxidized while nicotinamide adenine dinucleotide (NAD) is catalyzed to NADH by the glucose-6-phosphate dehydrogenase enzyme. In a final analysis, the liposomes were analyzed for their cholesterol content using the LabAssay™ Cholesterol Assay Kit (FujiFilm Wako Chemicals Europe GmbH; Neuss, Germany).

### 2.4. In Vitro Studies

#### 2.4.1. Cell Culture and Reagents

Human U-87 MG GBM cells and murine bEnd.3 brain endothelial cells were obtained from the American Type Culture Collection (ATCC; HTB-14 and ATCC CRL-2299, Manassas, VA, USA). Dulbecco’s modified eagle medium (DMEM) with different glucose concentrations 4.5 g/L (high), 1 g/L (low), or 0 g/L was used as culture medium. The culture media were each supplemented with 10% (*v*/*v*) FBS, 100 U/mL penicillin, 0.1 mg/mL streptomycin, and 0.1% (*v*/*v*) non-essential amino acids (NEAA).

#### 2.4.2. Cell Staining Assay

Cells were seeded in 24-well plates with a cell count of 6 × 10^4^ cells/well and incubated for 24 h at 37 °C and 10% CO_2_. DMEM with the above-mentioned different glucose concentrations was used as culture medium. After a washing step with Dulbecco’s phosphate saline (DBPS), the cells were detached with Accutase^®^. After transferring the cells to a FACS tube, a cell pellet was formed by centrifugation at 150× *g* for 5 min and resuspended in FACS buffer (DPBS with 5% (*v*/*v*) FBS). Subsequent blocking of non-specific Fc-mediated antibody interactions was performed by incubation for 20 min at room temperature with anti-mouse (Ab93) or anti-rat CD16/CD32 antibody (D34-485) or with human Fc-block binding inhibitor (Fc1, all antibodies from BD Biosciences, Franklin Lakes, NJ, USA). After removal of the supernatant, the cells were incubated with 4% (*v*/*v*) paraformaldehyde for 20 min at room temperature on the shaker. After washing twice with FACS buffer, the cells were permeabilized by dropwise addition of 2–3 drops of 100% ice-cold methanol and subsequent incubation for 5 min at room temperature on the shaker.

After washing again three times with FACS buffer, the cells were stained by adding 100 µL of a 1:200 dilution of the phycoerythrin (PE)-coupled GLUT1 antibody (EPR3915, abcam, Cambridge, UK) or PE-coupled 5-HT_7_ antibody (AA 405-433, antibodies-online GmbH, Aachen, Germany) in FACS buffer. Staining is carried out by incubation in a refrigerator under light-protected conditions for 30 min. The cells are then resuspended in FACS buffer, washed three times and finally resuspended with 500 µL FACS buffer. The mean fluorescent intensities (MFI) were determined using a flow cytometer (LSR II, BD Biosciences, Franklin Lakes, NJ, USA) by measuring 10^4^ single-cell events.

#### 2.4.3. Liposomal Uptake Assay

Cells were seeded in 48-well plates with a cell count of 3 × 10^4^ cells/well and incubated for 24 h at 37 °C and 10% CO_2_ under complete glucose deprivation. After aspiration of the culture medium, 90% of the well volume was replaced with culture medium. Subsequently, 10% of the well volume was added with the liposomal formulations to be tested, resulting in the final liposomal concentrations of 100 µM, 500 µM, and 1000 µM in order to test a broad range of concentrations to elucidate the underlying uptake kinetics and possible saturation limits. The necessary dilutions of the liposomal formulations to a concentration of either 10 mM, 5 mM, or 1 mM were prepared in advance with the culture medium. In addition, 2-NBDG (Cayman, Ann Arbor, MI, USA) was added as a positive uptake control in two different concentrations of 50 µM and 150 µM under starving conditions in separate wells. Incubation was carried out for 1 h or 3 h in an incubator at 37 °C and 10% CO_2_.

Cellular uptake of the liposomes was stopped by aspiration of the medium and treatment of the cells with ice-cold DPBS. To detach the cells, trypsin-EDTA (BioWest S.A.S, Nuaillé, France) diluted 1:10 with DBPS was added and inactivated with cold FACS buffer. The samples were then transferred to FACS tubes and washed three times with FACS buffer. The mean fluorescent intensities (MFI) were determined using the BD LSR II flow cytometer by measuring 10^4^ single-cell events.

A coordinate transformation (*x* → −*x*; *y* → −*y*) and an exponential approximation was iterated until a coefficient of variation of *r*^2^ = 1 was achieved, using *S* as the iteration parameter; please see Equation (1). From this approximation, the cellular saturation *S* and the saturation half-time *t*_1/2_ resulted, which were used for data interpretation. As shown in Equation (1), this biophysical model according to Ashraf et al., 2020 [53], assumes an exponential convergence of the time-dependent uptake *I*(*t*) to a saturation limit *S*.

(1) $$I\left(t\right)=S\left(1-exp\left(-\frac{t}{k}\right)\right)\;with\;k=t_{1/2}ln(2)$$

#### 2.4.4. Cytotoxicity Assay

Cells were seeded in 96-well plates with a cell count of 2 × 10^4^ cells/well and incubated for 48 h at 37 °C and 10% CO_2_. After 48 h of incubation, the medium was changed, whereby only 90% of the well volume was replaced with fresh medium. The remaining 10% is replaced with the corresponding dilutions of the liposomal formulations. The liposomal formulations were added for the final concentrations of 100, 500, and 1000 µM and incubated for 3 h according to the uptake assays. Cell viability was determined by adding 10 µL of alamarBlue™ HS (Thermo Fisher Scientific, Waltham, MA, USA) reagent, measuring the reducing power of the living cells. It was incubated for 2 h to measure the absorbance on the multiplate reader (wavelength 570 nm and reference wavelength 600 nm, Sunrise, Tecan, Männedorf, Switzerland).

### 2.5. Statistical Analysis

Data are presented as means ± SD. One-way or two-way ANOVA with subsequent post hoc tests (Tukey’s or Šidák multiple comparisons) were used to compare across three or more separate groups. A *p*-value < 0.05 was considered significant. An adapted Student’s *t*-test was performed to analyze the trend during liposomal storage.

## 3. Results

### 3.1. Characterization and Stability of Liposomes

The results of the size and size distribution characterization (Z-Average and PdI) of the liposomes are summarized in Table 2. All liposomal formulations showed a Z-Average (Z-Ave) of around 100 nm. In addition, all liposomes had a very small size distribution, which can be seen from the low PdI values < 0.1, except for GLU with a PdI of 0.159 ± 0.032.

The glucose concentrations of the functionalized formulations GLU and APG were determined using the glucose hexokinase assay kit. The GLU liposomes had a glucose concentration of 0.279 ± 0.009 mg/mL, and APG had a glucose concentration of 0.154 ± 0.010 mg/mL. Employing the determined cholesterol concentrations, the conjugation efficiency and the molar percentage of the APG post-conjugate as well as the molar percentage of the incorporated DSPE-PEG5k-GLU pre-conjugate were calculated. The conjugation efficiency for APG is 81.40%, and the recalculated molar percentage is 5.85% for the APG ligand, while the molar percentage of the glucose pre-conjugate GLU is 10.91 mol%.

**Figure 2:**
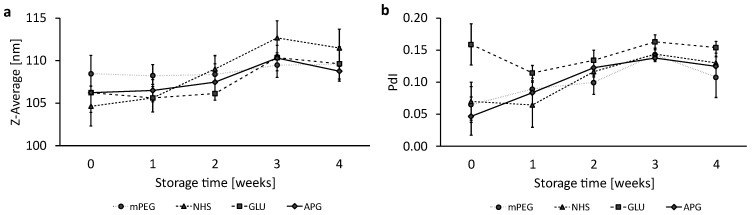
Liposomal stability over a storage period of 4 weeks at 4 °C. A representation of (**a**) the Z-Average and (**b**) the PdI. The dots represent the mean values with the standard deviation as error bars, n = 3.

The stability of the liposomal formulations was investigated for four weeks while stored at 4 °C under light-protected conditions. Figure 2a shows the evolution of size over 4 weeks; please note that the *y*-axis only shows a size range of 100–115 nm. Linear regressions of the size evolution of the four liposomal formulations were analyzed by a Student’s *t*-test; please see Supplementary Figure S1. Only NHS liposomes are shown to have a significant size increase. All other formulations showed non-significant fluctuations in particle size over 4 weeks.

The polydispersity index (PdI) served as a further characteristic for assessing liposomal stability. As presented in Figure 2b, NHS, APG, and mPEG showed a similar increase in polydispersity, whereby the increase in the PdI for NHS and APG is significant.

The individual graphs of the trend analysis for the Z-Ave (Figure S1) and the PdI (Figure S2) as well as a summarizing data table (Table S1) can be found in the Supplementary Information.

In addition, the average number of glucose molecules per liposome was estimated by forming the ratio of the number of glucose molecules per unit volume *N*_Glucose_, and the number of liposomes per unit volume *N*_Liposomes_ correlated with the liposomal size and lipid concentration. For the GLU liposomes, an average number of ~9650 glucose molecules per liposomes was calculated, whereas for the APG liposomes, an average number of ~6040 glucose molecules per liposome was estimated.

### 3.2. Expression Levels of GLUT1 in Endothelial and Cancer Cells under Different Glucose Concentrations

**Figure 3:**
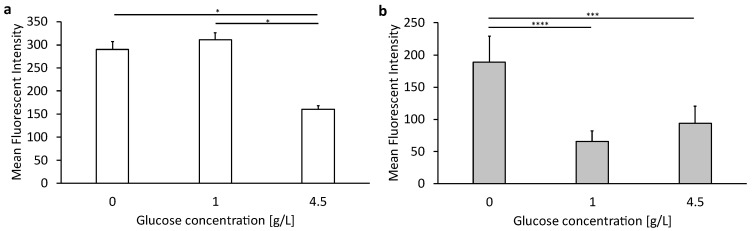
Expression levels of GLUT1 in bEnd.3 cells and U-87 MG cells after culturing with different glucose concentrations in media. (**a**) A representation of target expression in bEnd.3 cells and (**b**) U-87 MG cells. The bars represent the mean values with the standard deviation as error bars. Statistical analysis: two-way ANOVA followed by Tukey’s multiple comparison test. * *p* < 0.05, *** *p* < 0.001, **** *p* < 0.0001; n = 3.

Figure 3 shows the expression of the glucose 1 transporter (GLUT1) in murine bEnd.3 brain endothelial cells (Figure 3a) and human U-87 MG glioblastoma cells (Figure 3b) at different glucose concentrations in the culture medium. Expression was determined by the staining of the transporter using the dye-coupled antibody EPR3915 and subsequent flow cytometric analysis.

As shown in Figure 3a, the bEnd.3 cells show the highest expression of GLUT1 at 0 or 1 g/L glucose in the medium, with no significant difference. The GLUT1 expression is, however, significantly lower when cultivated under high glucose conditions with 4.5 g/L. In contrast, human U-87MG GMB cells showed the lowest expression when cultured with low or high glucose conditions in the cell culture medium, with no significant difference. Cells cultured under glucose deprivation showed the highest expression.

The corresponding data table can be found in the supplements (Table S2).

### 3.3. Cellular Uptake of Succinimide- or Glucose-Modified Lipsoomes in Endothelial Cells

**Figure 4:**
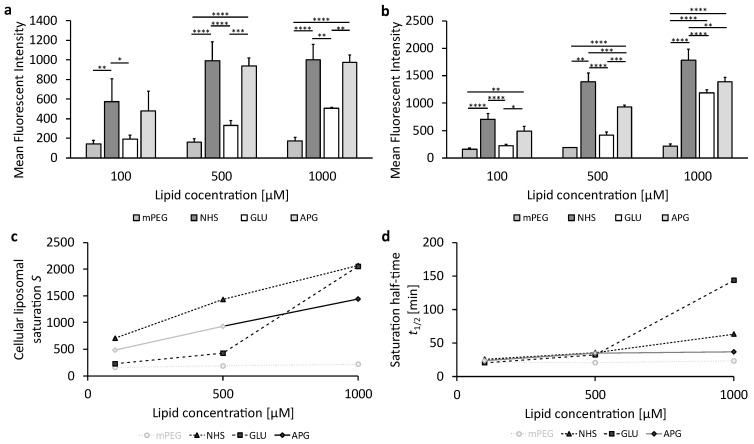
The uptake of glucose-modified liposomes in bEnd.3 cells under complete glucose deprivation conditions. (**a**) Liposomal uptake in bEnd.3 cells after 1 h and (**b**) 3 h of treatment. The bars represent the mean values with the standard deviation as error bars. Statistical analysis: two-way ANOVA followed by Tukey’s multiple comparison test. * *p* < 0.05, ** *p* < 0.01, *** *p* < 0.001, **** *p* < 0.0001; n = 3. (**c**) The cellular liposomal saturation and (**d**) the corresponding saturation half-time t_1/2_ calculated according to Equation (1). In those cases where the exponential regression only produced *r*^2^ < 1, the mean fluorescence intensity (MFI) at 3 h is used as an approximation of the cellular saturation *S*, and these data are shown in faint grey. The data points in faint gray in (**d**) are calculated using approximate values for the exponential fit.

To investigate the ability of liposomes or 2-NBDG as a positive control concerning their internalization in murine bEnd.3 brain endothelial cells under complete deprivation of glucose (0 g/L), the cells were incubated with increasing liposomal concentrations. Figure 4a,b show the liposomal uptake at 1 h or 3 h (cf. corresponding data in Table S3 in the supplements). Figure 4c represents the cellular liposomal saturation *S*, and Figure 4d shows the saturation half-time *t*_1/2_. The supplementary graphs summarizing the plots of the mean fluorescence intensities (MFI) over time and the exponential fits for the various liposomal formulations can be found in Figure S3 in the supplements; values for the cellular liposomal saturations *S* and saturation half-time *t*_1/2_ are summarized in Table S4. Figure S3g contains exemplarily the cellular liposomal saturation *S* and the cellular saturation half-time *t*_1/2_ as red lines.

As can be seen in Figure 4a,b, the measurement data of cellular uptake at 1 h and 3 h displayed as bar plots provide the impression of complex relationships of significant or insignificant differences. These are noted by the interconnecting lines with stars for the different levels of significance. At liposomal concentrations of 100 µM and 500 µM, PEGylated (mPEG) and glucose-modified (GLU) liposomes show low uptake, while succinimide-modified (NHS) and 4-aminophenyl β-D-glucopyranoside-modified (APG) liposomes exhibit significantly higher cellular uptake.

The uptake of mPEG liposomes remains low at the highest liposomal concentration of 1000 µM (Figure 4b). In contrast, GLU and NHS liposomes have a more pronounced increase in cellular uptake with increasing liposomal concentration, while APG liposomes increase much less at this high concentration.

Cellular uptakes of the four liposomal samples can, however, only be compared and understood mechanistically by observing the exponential evolutions converging to their specific saturation limits *S* at different speeds, characterized by the saturation half-times *t*_1/2_ as shown in Figure 4c,d. NHS liposomes have the highest cellular saturations *S* for all incubation concentrations *c*, which are doubled when *c* is increased from 100 µM to 500 µM and show a threefold increase in *S* upon a 10-fold increase in *c* from 100 µM to 1000 µM. The cellular saturation *S* of APG liposomes increases parallel to that of NHS liposomes, but saturation for APG is at around 67% of that for NHS at all *c*. The saturation half-time *t*_1/2_ of APG liposomes is apparently very small, leading to the fact that cellular uptake is almost complete at 1 h and 3 h, leading to the fact that the exponential regressions cannot be fitted very precisely with *r*^2^ < 1. In such cases, we use the MFI data at 3 h as a close approximation to *S*, indicated in Figure 4c,d by fainted symbols and interconnecting lines.

In contrast to the parallel increases in NHS and APG liposomes, GLU liposomes show very low cellular saturations *S* at both 100 and 500 µM, being only about 30% of those of NHS liposomes. Surprisingly, *S* increases strongly for the highest *c* of 1000 µM so that both GLU liposomes have an *S* almost as high as NHS liposomes. Different characteristics can be found for mPEG liposomes which can only be fitted with an *r*^2^ < 1. Therefore, the fluorescence intensity at 3 h is used as *S*, showing extremely low cellular saturations of only 22%, 13%, and 11% of the saturation for NHS for increasing concentrations from 100 µM to 500 µM and 1000 µM, respectively. Thus, increasing *c* has only a very minor effect on *S* for mPEG, which is only increased by 38% when *c* is increased 10-fold from 100 µM to 1000 µM.

Figure 4d shows the saturation half-times *t*_1/2_ for the four liposomal samples. Since the exponential regressions for mPEG are unprecise (*r*^2^ < 1), the results are not sufficiently quantifiable but indicate short half-times for all concentrations. Surprisingly, the half-times of all three other samples, NHS, GLU, and APG, are uniform for the lower concentrations 100 µM and 500 µM but diversify for 1000 µM. Here, APG liposomes are fastest in reaching the saturation half-time, followed by NHS, with GLU being slowest in reaching the saturation half-time, needing almost 4 times longer to reach *t*_1/2_.

For the cellular uptake of 2-NBDG as a positive control, shown in Supplementary Figure S4, with corresponding data in Table S5, the uptake of 2-NBDG was found to be both time and concentration dependent.

### 3.4. Cellular Uptake of Succinimide- or Glucose-Modified Lipsoomes in Glioblastoma Cells

**Figure 5:**
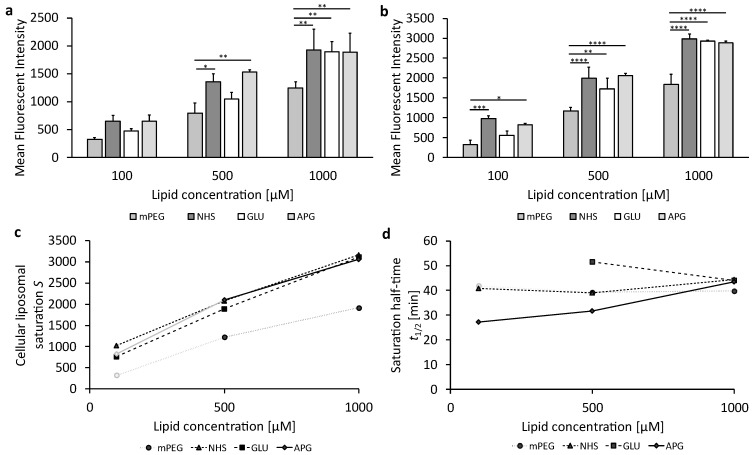
The cellular uptake of glucose-modified liposomes for U-87 MG cells under starving conditions (0 mg/mL glucose in cell medium). (**a**) Liposomal uptake in U-87 MG cells after 1 h and (**b**) 3 h of treatment. The bars represent the mean values with the standard deviation as error bars. Statistical analysis: two-way ANOVA followed by Tukey’s multiple comparison test. * *p* < 0.05, ** *p* < 0.01, *** *p* < 0.001, **** *p* < 0.0001; n = 3. (**c**) Cellular liposomal saturation *S* vs. lipid concentration as calculated according to Equation (1). (**d**) Saturation half-time t_1/2_ according to Equation (1) vs. lipid concentration. In those cases where the exponential regression only produced *r*^2^ < 1, the mean fluorescence intensity (MFI) at 3 h is used as an approximation to the cellular saturation *S*, but these data are shown in faint grey. The saturation half-time value for GLU liposomes at 100 µM was discarded due to the insufficient accuracy of the regression.

The cellular uptake of different liposomal formulations under complete glucose deprivation was studied also for the human glioblastoma cell line U-87 MG at three different lipid concentrations (100 µM, 500 µM, and 1000 µM) for two incubation periods (1 h and 3 h). The results are shown in Figure 5a,b and the corresponding data in Table S6 in the supplements. Figure 5c,d are similar to Figure 4c,d, representing the cellular liposomal saturation and the saturation half-time with the corresponding Supplementary Figure S5, and data in Table S7.

Contrary to the murine endothelial bEnd.3 cells, human U-87 MG glioblastoma cells show almost uniform cellular uptake. At the lowest concentration of 100 µM at 1 h, cellular uptake of mPEG liposomes is not yet significantly lower than all of the other three liposomal coatings (NHS, GLU, and APG); this only occurs for higher concentrations and longer incubation. Although GLU liposomes have lower MFI at 100 µM and 500 µM, this subtle distinction is not significant and vanishes for the highest concentration.

The mechanisms behind these raw data are disclosed by the saturations of cellular uptake and their respective half-times as shown in Figure 5c,d. As can be seen, the saturations are almost identical for the three surface coatings: NHS, GLU, and APG; only mPEG liposomes have substantially lower saturations. The uniformity of the three coatings (NHS, GLU, APG) regarding their cellular saturation is contrasted by a strong diversification regarding their half-times for the lower concentrations of 100 µM and 500 µM, as shown in Figure 5d. Clearly, GLU is slowest in cellular uptake (at 500 µM, not determinable for 100 µM), and APG is fastest, while NHS and mPEG are in between these extremes. Surprisingly, all four liposomal coatings produce almost identical half-times at the highest concentration of 1000 µM. In total, the human glioblastoma cell line U-87 MG has quite distinct characteristics regarding the cellular uptake of PEGylated liposomal with differences in the distal polymer ends: while saturation is identical for all liposomal coatings except for mPEG, the saturation half-times are clearly diversified at low and intermediate concentrations but unify at the highest concentration.

### 3.5. Expression Levels of 5-HT7 in Endothelial and Cancer Cells under Normal Cultivation Conditions

To evaluate the expression of the 5-HT_7_ receptor, its staining was performed analogously to GLUT1, using a labeled antibody, the phycoerythrin (PE)-coupled 5-HT_7_ antibody AA 405-433, as described in Section 2.4.2. The cellular staining of the receptor was performed under normal glucose conditions of 4.5 g/L glucose in the culture medium. The comparison of treated cells stained with antibody versus unstained cells showed the significant expression of the 5-HT_7_ receptor in both bEnd.3 cells (MFI 3631.50 ± 586.90) and U-87 MG cells (MFI 5910.40 ± 486.70).

### 3.6. Cellular Viability after Treatment with the Liposomal Formulations

**Figure 6:**
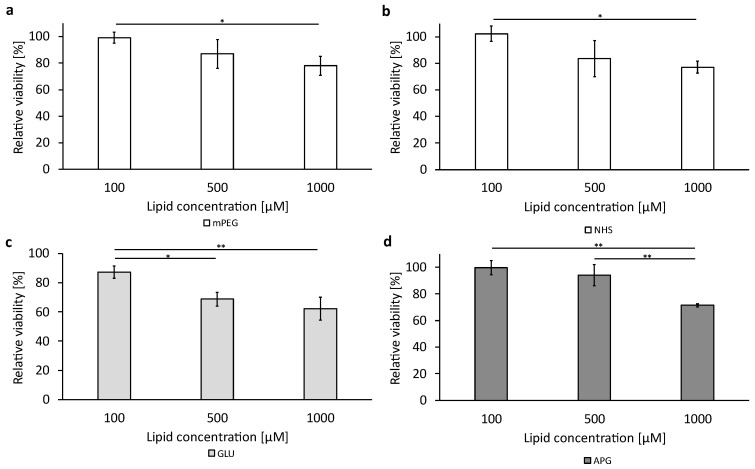
Liposomal cytotoxicity on bEnd.3 cells using alamarBlue™ HS reagent. A representation of all different tested formulations: (**a**–**d**). The bars represent the mean values with the standard deviation as error bars. Statistical analysis: one-way ANOVA followed by Tukey’s multiple comparison test. * *p* < 0.05, ** *p* < 0.01; n = 3.

**Figure 7:**
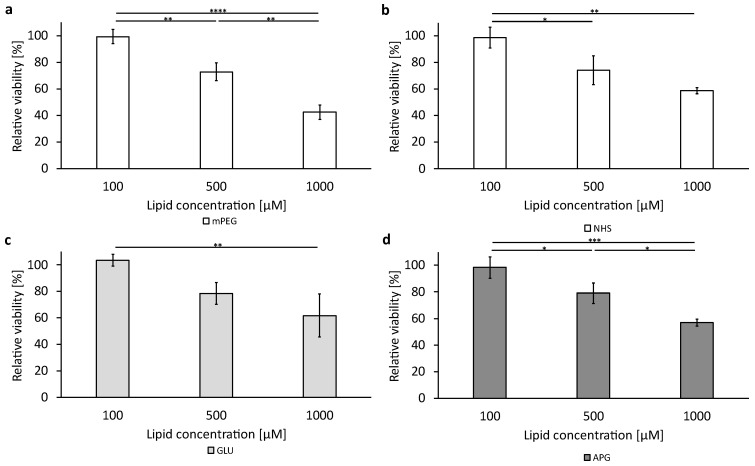
Liposomal cytotoxicity on U-87 MG cells using alamarBlue™ HS reagent. A representation of all different tested formulations: (**a**) mPEG; (**b**) NHS; (**c**) GLU; (**d**) APG. The bars represent the mean values with the standard deviation as error bars. Statistical analysis: one-way ANOVA followed by a Tukey multiple comparison test. * *p* < 0.05, ** *p* < 0.01, *** *p* < 0.001, **** *p* < 0.0001; n = 3.

To assess the cytotoxic effect of the basic liposomal formulations and the functionalized formulation without encapsulated cytotoxic active pharmaceutical ingredient (API), a cell viability assay was performed as described in Section 2.4.4. The data are shown in Figure 6 and Figure 7 for the bEnd.3 cells and the U-87 MG, respectively, with subgraphs a to d showing the four liposomal formulations tested at the three concentrations (100 µM, 500 µM, and 1000 µM). Corresponding data tables can be found in the Supplementary Tables S9 and S10.

The bEnd.3 cells (Figure 6) showed a reduction in viability of around 70% after 3 h of the incubation of the cells with liposomes at the highest concentration of 1000 µM, except the GLU liposomes (Figure 6c), where the viability was reduced to 62.62% ± 7.91.

The U-87 MG cells showed a stronger reduction in viability at the highest concentration tested compared to the bEnd.3 cells. Viability decreased to around 60% for all formulations except for the mPEG liposomes (Figure 7a), with a viability of 42.42% ± 5.26 when incubated with 1000 µM for 3 h.

## 4. Discussion

Glucose as a ligand has attracted great interest concerning various carrier systems aiming for active targeting, as the glucose transporter is mainly expressed in the endothelial cells of the CNS [54]. This is consistent with the results in Figure 3, which show that GLUT1 expression is generally higher in bEnd.3 endothelial cells compared to expression in human glioblastoma cells U-87 MG. The staining of GLUT1 was performed to determine whether hypoglycemic conditions promote the expression of GLUT1 in bEnd.3 and U-87 MG cells, as described by Simpson et al., 1999, among others [55]. The results in Figure 3 show a significant upregulation of GLUT1 under hypoglycemic conditions with complete glucose deprivation (0 g/L glucose) in the medium. bEnd.3 cells are more sensitive to changes in the glucose concentration in the medium. Already, 1 g/L glucose in the medium causes a significant upregulation of GLUT1 compared to its expression at 4.5 g/L, while U-87 MG cells do not express more GLUT1 when glucose is reduced from 4.5 g/L to 1.0 g/L. One possible reason for the less sensitive reaction of the tumor cells U-87 MG is the fact that tumor cells have an adapted metabolism. Thus, mitochondria in tumor cells can use lactate as fuel for biochemical reactions to enable cell growth [56,57].

GLUT1 is present on both the luminal and abluminal membranes of BCECs [58], so glucose deprivation can increase the localization of GLUT1 at the luminal plasma membrane by up to 50% [55]. Thus, GLUT1 moves from the cell interior to the plasma surface depending on the glucose demand in the brain. Generally, endothelial cells have very high glycolytic rates, similar to many cancer cells [59]. Nevertheless, the higher liposomal uptake in U-87 MG cells (Figure 5) compared to liposomal uptake in endothelial cells bEnd.3 (Figure 4) indicates that liposomal uptake is not merely triggered by environmental glucose concentration but also by cell-specific metabolic rates.

There is not only a higher uptake of liposomes in the tumor cell line U-87 MG than in the endothelial cells bEnd.3, which is in contrast to the GLUT1 cell surface concentration, but also a fundamentally different uptake behavior between the two cell lines. For example, the endothelial cells bEnd.3 differentiate liposomes concerning the cellular uptake according to their surfaces, as the cellular saturations at low and medium concentrations are very different between the individual liposomal formulations (Figure 4c). The most important finding of this study is that the succinimide residue (NHS) proves to have a much higher liposomal uptake saturation than APG- and GLU-coated liposomes, which makes it a very promising and so far largely unexplored candidate for BBB transfer and brain cancer therapies.

Succinimides are widely studied due to their extensive pharmacological applications. Recent studies showed potential applications of succinimide structural analogs as a serotonin 5-hydroxytryptamine receptor ligand [60]. However, it should be noted that to date, there are no publications on the use of succinimides as ligands of drug delivery systems. The 5-HT_7_ receptor is highly expressed in the CNS [61], which was also demonstrated in our staining experiments. The binding of succinimide to the 5-HT_7_ receptor leads to clathrin-mediated endocytosis [62]. The expression of the 5-HT_7_ receptor in the U-87 MG cells is increased compared to the bEnd.3 cells, which is shown by the mean fluorescent intensities of around 4000 and 6000, respectively. Despite the higher expression of the 5-HT_7_ receptor, the uptake of NHS liposomes in U-87 cells is not favored over the other modified liposomes. The 5-HT_7_ receptor is one of many subtypes of serotonin receptors, so cellular uptake can also occur through other receptor subtypes. Since most 5-hydroxytryptamine receptors are also G-protein coupled receptors, it is of interest for future studies to investigate further details in the uptake mechanism of NHS liposomes using uptake inhibition assays.

In contrast to the uptake in bEnd.3 cells, there is almost no difference in cellular saturation between liposomes with different distal ends of the PEG5k chain in the U-87 MG cells (Figure 5c). When looking at the saturation half-time, it can be seen that the “speed of uptake” in bEnd.3 cells is uniform at low concentrations and starts to differentiate at high lipid concentrations (Figure 4d), whereas in U-87 MG cells, it is the opposite. For tumor cells such as the U-87 MG cells, the rate of liposomal uptake differs greatly at low and medium lipid concentrations but is nearly of the same speed for all liposomal formulations at the highest lipid concentration (Figure 5d). The uniformity of the liposomal uptake saturation for all three ligands NHS, GLU, and APG makes it very likely that uptake in U-87 MG cells is not very strongly affected by ligand-specific transporters. This aspect needs to be explored in more detail to find clear proof on liposomal uptake mechanisms in glioblastoma cells.

It has to be assumed that the GLUT1 only serves as a recognition pattern for the liposomes, whereas the uptake of liposomes is achieved by other endocytotic mechanisms like receptor-mediated endocytosis (RME) by, e.g., the 5-HT_7_ receptor. This assumption is obvious, as, e.g., GLUT1 has a cavitation volume (exofacial occluded) of 4385 Å^3^ [63], whereas a liposome of 110 nm has a volume of roughly 700 × 10^6^ Å^3^. Jiang et al., 2014, showed that both caveolae-mediated and clathrin-mediated endocytosis are involved in the cellular uptake of D-Glucose-decorated PEG-PTMC co-polymeric nanoparticles by glioma cells [64]. This example demonstrates that only the endocytosis mechanisms can lead to liposomal intracellular uptake.

In general, mPEG liposomes show the lowest intracellular uptake regardless of the cell line or incubation time. This is in line with our previous study [46], where we were able to show the intracellular uptake of mPEG liposomes as an energy-depending process, as the uptake nearly dropped to zero when incubation is performed at 4 °C. In addition, the intracellular uptake of liposomes in our current study is confirmed by the increasing cytotoxicity, with increasing liposomal uptake interfering with the intracellular reducing activity as an indicator of their viability.

GLUT1 binds glucose molecules primarily through weak interactions, such as hydrogen bonds and hydrophobic effects [65]. Therefore, the binding of a single glucose molecule is not strong enough to retain the nanocarrier in the blood flow of the organism. To achieve a strong retention of nanocarriers on the BCECs by GLUT1 binding, multivalent interactions by a high surface density of glucose molecules on the liposomal surface are preferable [66,67]. Qin et al., 2010, were able to prove the hypothesis that a higher surface density of glucose on the liposomal surface causes a higher cellular uptake [68]. They showed that a liposomal formulation with almost 33 mol% of a cholesterol–glucose conjugate led to the highest cellular uptake.

In our study, we achieved increased uptake with glucose-modified liposomes having 5.85 mol% glucose on the surface of APG liposomes and 10.91 mol% on GLU liposomes. For both compositions of the liposomes, it was intended to include 5 mol% glucose. For this aim, liposomes were produced based on an assumed molecular weight of an approx. 5 kDa PEG-chain + 745 Da (DSPE). After the fabrication of the liposomes, glucose analytics in comparison to the cholesterol analytics showed the mentioned excess glucose contents of 5.9 mol% and 10.9 mol%, respectively. We therefore have to conclude that the difference is due to an inaccurate specification of the glucose-conjugating PEG-DSPE anchors’ molecular weights by the manufacturer. This inaccuracy is caused by the polydisperse PEG chain length of DSPE-PEG5k-GLU or DSPE-PEG5k-NHS.

Despite the very high glucose content of the GLU liposomes, APG liposomes showed higher uptake in bEnd.3 cells at low and medium concentrations despite the lower glucose molar percentage. This could probably be the result of two counteracting factors: (1) The unconjugated succinimide residues, as the conjugation efficiency is at around 80%. The free NHS groups with a surface density of approx. 1.5 mol% might cause the higher uptake of APG liposomes despite the higher glucose surface concentration of the GLU liposomes and (2) the conjugation position for the covalent binding of 4-aminophenyl β-D-glucopyranoside, which occurs at the C1 position. According to Barnett et al., 1973, covalent binding via the C-6 position of the glucose is preferable, as this preserves its ability to bind to GLUT1 [69]. The interaction of glucose with GLUT1 is essentially based on the hydroxyl groups in positions C-1, C-3, and C-4 [69]. In our study, the APG liposomes increased cellular uptake despite conjugation via the C-1 position. This might be the result of the unconjugated succinimide residues, which overcompensate the disadvantage of the APG liposomes in their ligand function with the GLUT1 receptor, at least in the low and medium liposome concentration range (100 µM and 500 µM). However, at the highest concentration of 1000 µM, GLU liposomes are superior to APG liposomes, which indicates that not only do NHS residues contribute to the selective cellular uptake in bEnd.3 cells but also glucose moieties.

## 5. Conclusions

In conclusion, essential differences exist in the uptake speed and saturation of liposomal formulations, which are both cell specific and specific to the surface coatings of nanoparticles. Liposomes containing a coating of 5 mol% of mPEG clearly have the lowest cellular uptake saturation. At the same time, these particles have the strongest impact on cellular viability measured by their reducing activity. Viability goes down to 78% and 42%, respectively, for bEnd.3 and U-87 MG cells incubated with 1000 µM lipid for 3 h, compared to their viabilities without liposomal uptake. In this comparison, liposomal uptake saturation is 7.8 times higher in the glioblastoma cells than in the endothelial cells. For all other liposomal coatings, viability is not lower than 57% even though uptake saturation for NHS, GLU, and APG liposomes is 60% higher than for mPEG liposomes, proving the much higher tolerability of these surface coatings for cell viability along with much higher uptake saturations. These findings may also provide new aspects to the frequently discussed PEG dilemma.

For practical applications, liposomal concentrations in the endothelium or tumor microenvironment will be much lower and clearly without impact on viability. For future clinical use in the targeting of brain tumors, it is most relevant that at a lipid concentration of 100 µM, succinimide-coated liposomes achieve 3.2 and 4.2 times higher cellular uptake in endothelial cells than the glucose coatings GLU and APG, respectively. We therefore expect a great potential for future clinical perspectives in exploring and developing the potential of succinimide- or serotonin-derived ligands in their interactions with the serotonin receptor family to enhance the BBB transfer of targeted therapies.

## Figures and Tables

**Table 1:** Composition of liposomal formulations.

Sample Nomenclature	mPEG	NHS	GLU	APG
Components	Molar Ratio [%]			
DPPC	54.9	49.9	49.9	49.9
Cholesterol	40	40	40	40
DSPE-PEG2k	5	5	5	5
DSPE-PEG5k-NHWS	-	5	-	-
DSPE-PEG5kGLU	-	-	5	-
DSPE-PEG5k-APG	-	-	-	5
DiD	0.1	0.1	0.1	0.1

**Table 2:** The summary of the particle size, the polydispersity index, and the Zeta potential of the tested liposomal formulations.

Liposomes	Z-Average [nm]	PdI	Zeta Potential [mV]
mPEG	108.5 ± 2.1	0.065 ± 0.028	−3.1 ± 0.3
NHS	104.6 ± 2.3	0.070 ± 0.030	−4.5 ± 4.5
GLU	106.2 ± 1.6	0.159 ± 0.032	−5.4 ± 1.6
APG	106.2 ± 2.3	0.047 ± 0.030	−5.1 ± 1.9

## Data Availability

The data supporting this article have been included as part of the Supplementary Information.

## Supplementary Materials

The following supporting information can be downloaded at https://www.mdpi.com/article/10.3390/biomedicines12092135/s1. Figure S1: Trend analysis of the Z-Average of the liposomal formulations over a storage period of 4 weeks at 4 °C. Determination of significance of a linear regression using Student’s *t*-test. Figure S2: Trend analysis of the PdI of the liposomal formulations over a storage period of 4 weeks at 4 °C. Determination of significance of a linear regression using Student’s *t*-test. Figure S3: Plot of the mean fluorescence intensities over time (a, c, e and g) and the corresponding exponential fits (b, d, f and h) for the cellular uptake of the different liposomal formulations into the bEnd.3 cells, where. (a and b) represent the data for the mPEG liposomes, (c and d) for the NHS liposomes, (e and f) for the GLU liposomes and (g and h) for the APG liposomes. Figure S4: Cellular uptake of 2-NBDG in bEnd.3 cells for two different times of incubation (one and three hours). The bars represent the mean ± SD. Statistical analysis: one-way ANOVA followed by a two-sample *t*-test assuming equal variance, ** *p* < 0.01, *** *p* < 0.001, n = 3. Figure S5: Plot of the mean fluorescence intensities over time (a, c, e and g) and the corresponding exponential fits (b, d, f and h) for the cellular uptake of the different liposomal formulations into the U-87 MG cells, where. (a and b) represent the data for the mPEG liposomes, (c and d) for the NHS liposomes, (e and f) for the GLU liposomes and (g and h) for the APG liposomes. Figure S6: Cellular uptake of 2-NBDG in U-87 MG cells for two different times of incubation (one and three hours). The bars represent the mean ± SD. Statistical analysis: one-way ANOVA followed by a two-sample *t*-test assuming equal variance, *** *p* < 0.001, **** *p* < 0.0001, n = 3. Table S1: Overview of particle size (Z-Average) and polydispersity index (PdI) over a storage period of 4 weeks at 4 °C. Values are given as mean ± SD, n = 3. Table S2: Mean fluorescent intensities (MFI) for the GLUT1 staining at different glucose concentrations in bEnd.3 cells and U-87 MG cells, n = 3. Table S3: Mean fluorescent intensities (MFI) for the liposomal uptake in bEnd.3 endothelial cells, n = 3. Table S4: Calculated cellular liposomal saturation (*S*) and the corresponding saturation half-time (*t*_1/2_) for bEnd.3 cells. Table S5: Mean fluorescent intensities (MFI) for the uptake of 2-NBDG in bEnd.3 endothelial cells, n = 3. Table S6: Mean fluorescent intensities (MFI) for the liposomal uptake in U-87 MG glioma cells, n = 3. Table S7: Calculated cellular liposomal saturation (*S*) and the corresponding saturation half-time (*t*_1/2_) for U-87 MG cells. Table S8: Mean fluorescent intensities (MFI) for the uptake of 2-NBDG in U-87 MG glioma cells, n = 3. Table S9: Values of the relative viability of the bEnd.3 cells, n = 3. Table S10: Values of the relative viability of the U-87 MG cells, n = 3.
