# Supplementary material for: Elevated Cellular Uptake of Succinimide- and Glucose-Modified Liposomes for Blood–Brain Barrier Transfer and Glioblastoma Therapy
Source: Biomedicines. 2024 Sep 20;12(9):2135. doi: 10.3390/biomedicines12092135 (PMC11430759; doi:10.3390/biomedicines12092135)
Supplement: Supplementary file 1 [file biomedicines-12-02135-s001.zip › biomedicines-3191864-supplementary.pdf]

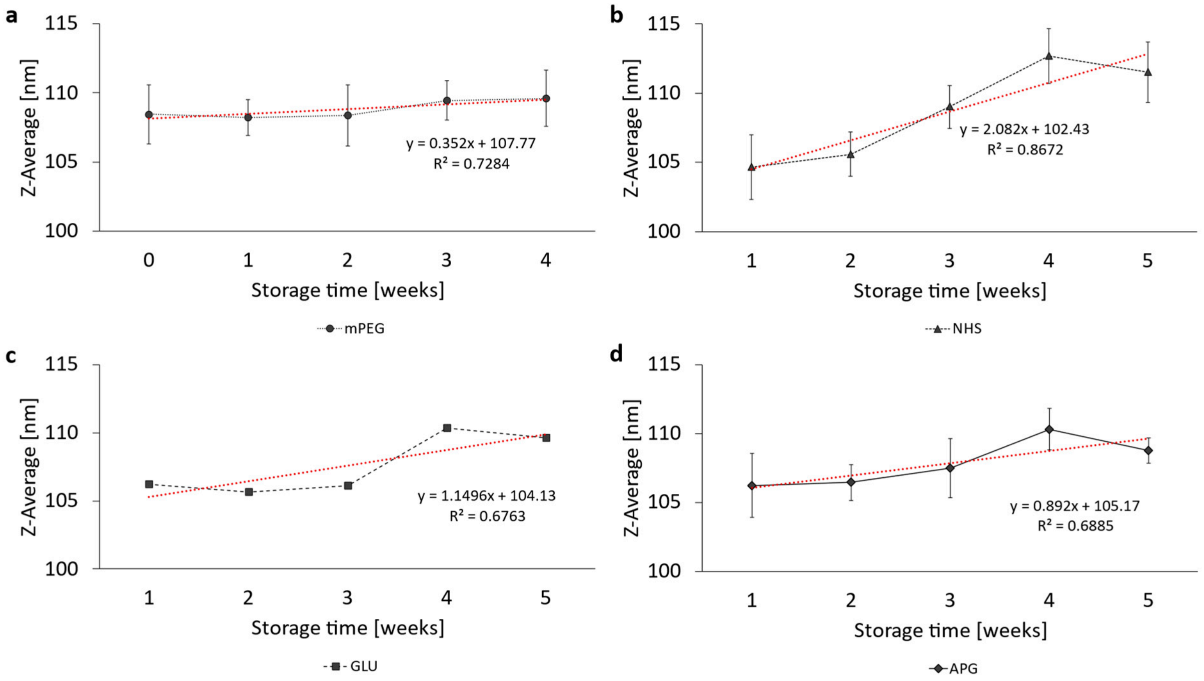

Supplementary Figure S1. Trend analysis of the Z-Average of the liposomal formulations over a storage period of 4 weeks at 4 °C. Determination of significance of a linear regression using Student's t-test.

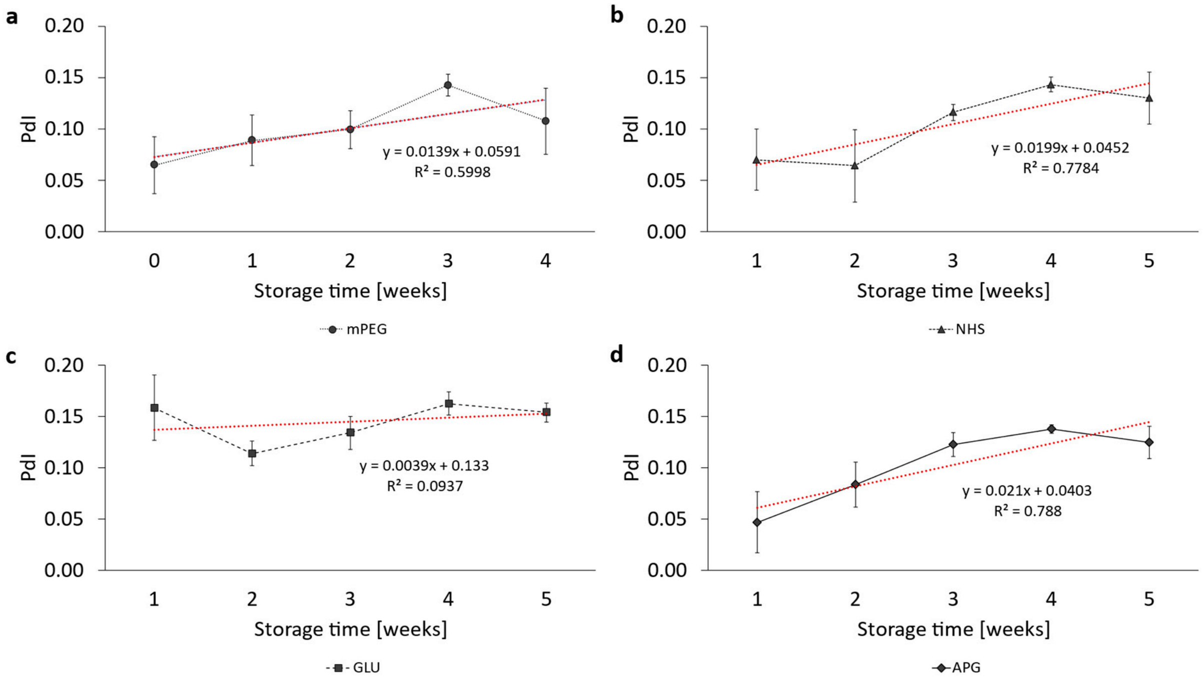

Supplementary Figure S2. Trend analysis of the PdI of the liposomal formulations over a storage period of 4 weeks at 4 °C. Determination of significance of a linear regression using Student's t-test.

Supplementary Table S1. Overview of particle size (Z-Average) and polydispersity index (PdI) over a storage period of 4 weeks at 4 °C. Values are given as mean ± SD, n = 3.

Type of liposome	Storage time [weeks]	Z-Average [nm]		PdI	
		Mean	SD	Mean	SD
mPEG	0	108.5	2.1	0.065	0.028
	1	108.2	1.3	0.089	0.025
	2	108.4	2.2	0.100	0.018
	3	109.5	1.4	0.143	0.011
	4	109.6	2.0	0.108	0.032
NHS	0	104.6	2.3	0.070	0.030
	1	105.6	1.6	0.065	0.035
	2	109.0	1.6	0.116	0.008
	3	112.7	2.0	0.144	0.007
	4	111.5	2.2	0.130	0.025
GLU	0	106.2	1.6	0.159	0.032
	1	105.6	3.0	0.114	0.012
	2	106.1	2.1	0.134	0.016
	3	110.3	2.4	0.163	0.011
	4	109.6	1.1	0.154	0.009
APG	0	106.2	2.3	0.047	0.030
	1	106.5	1.3	0.084	0.022
	2	107.5	2.1	0.123	0.012
	3	110.3	1.5	0.138	0.004
	4	108.8	0.9	0.125	0.016

Supplementary Table S2. Mean fluorescent intensities (MFI) for the GLUT1 staining at different glucose concentrations in bEnd.3 cells and U-87 MG cells, n = 3.

Glucose concentration [g/L]	bEnd.3 cells		U-87 MG cells	
	Mean	SD	Mean	SD
0	290.22	16.46	188.43	40.36
1	311.23	15.40	65.40	16.60
4.5	160.61	7.74	93.69	26.58

Supplementary Table S3. Mean fluorescent intensities (MFI) for the liposomal uptake in bEnd.3 endothelial cells, n = 3.

Type of liposome	Concentration [μM]	1 hour of incubation		3 hours of incubation	
		Mean	SD	Mean	SD
mPEG	100	144.65	34.47	158.62	25.46
	500	158.98	38.89	188.57	6.56
	1000	172.33	38.25	219.06	36.89
NHS	100	574.10	232.97	708.42	100.87
	500	990.56	194.42	1393.00	161.27
	1000	1000.46	157.48	1784.00	197.81
GLU	100	193.96	38.15	222.52	21.92
	500	331.29	49.19	419.26	50.79
	1000	508.56	7.95	1190.26	54.17
APG	100	481.26	203.10	485.63	93.48
	500	936.21	82.87	928.37	32.10
	1000	974.18	75.12	1389.09	78.37

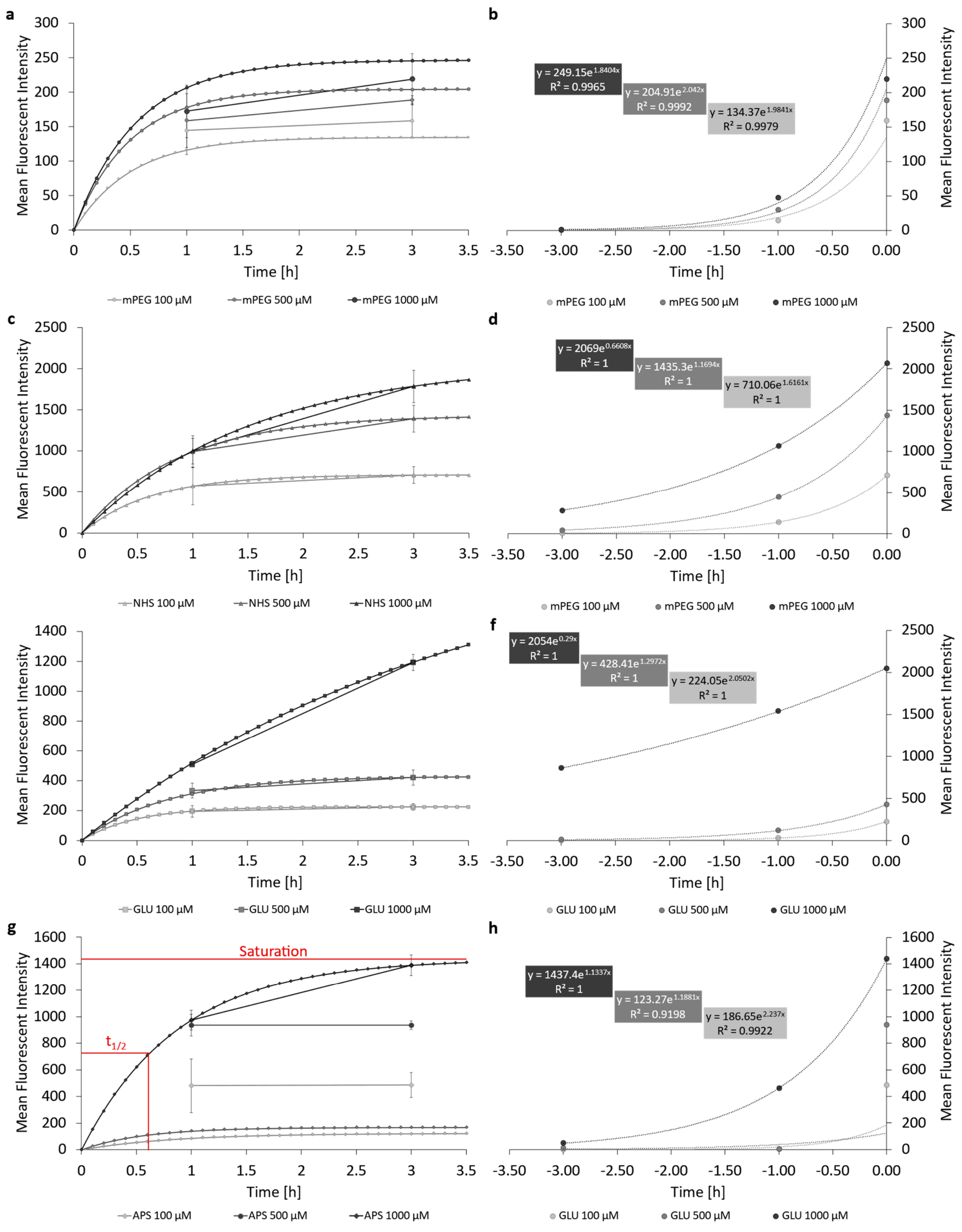

Supplementary Figure S3. Plot of the mean fluorescence intensities over time (a, c, e and g) and the corresponding exponential fits (b, d, f and h) for the cellular uptake of the different liposomal formulations into the bEnd.3 cells, where. (a and b) represent the data for the mPEG liposomes, (c and d) for the NHS liposomes, (e and f) for the GLU liposomes and (g and h) for the APG liposomes.

Supplementary Table S4. Calculated cellular liposomal saturation (*S*) and the corresponding saturation half-time ($t_{1/2}$) for bEnd.3 cells.

Type of liposome	Concentration [µM]	Fluorescence intensity at saturation conditions	time to reach half saturation conditions [min]
mPEG	100	134.4	21.0
	500	204.7	20.4
	1000	246.8	22.9
NHS	100	710.1	25.7
	500	1435.3	35.6
	1000	2069.0	62.9
GLU	100	224.1	20.3
	500	428.4	32.1
	1000	2054.0	143.4
APG	100	168.0	23.4
	500	123.3	35.0
	1000	1437.4	36.7

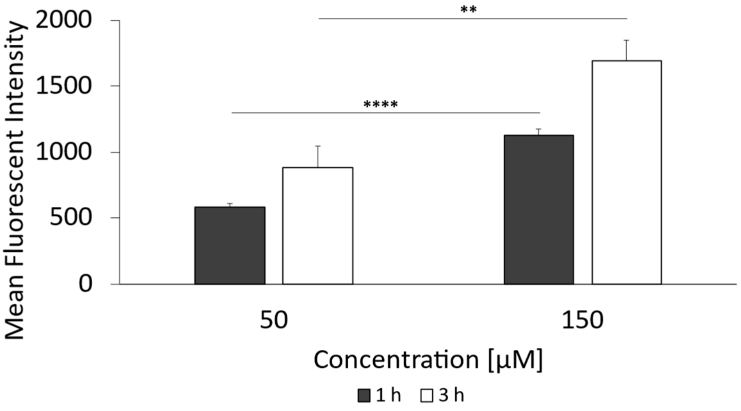

Supplementary Figure S4. Cellular uptake of 2-NBDG in bEnd.3 cells for two different times of incubation (one and three hours). The bars represent the mean ± SD. Statistical analysis: one-way ANOVA followed by a two-sample t-test assuming equal variance, **$p < 0.01$, ***$p < 0.001$, n = 3.

Supplementary Table S5. Mean fluorescent intensities (MFI) for the uptake of 2-NBDG in bEnd.3 endothelial cells, n = 3.

	Concentration [µM]	1 hour of incubation		3 hours of incubation	
		Mean	SD	Mean	SD
2-NBDG	50	585.59	22.50	879.77	164.83
	150	1123.81	50.83	1693.54	157.34

Supplementary Table S6. Mean fluorescent intensities (MFI) for the liposomal uptake in U-87 MG glioma cells, n = 3.

**Type of liposome**	**Concentration [μM]**	**1 hour of incubation**		**3 hours of incubation**	
		**Mean**	**SD**	**Mean**	**SD**
**mPEG**	100	325.75	35.59	321.18	121.93
	500	797.52	178.02	1170.32	94.33
	1000	1243.29	116.07	1833.00	262.14
**NHS**	100	655.56	98.69	977.80	67.27
	500	1360.59	142.71	1991.61	275.55
	1000	1925.82	373.49	2978.00	122.75
**GLU**	100	476.21	42.78	563.73	111.30
	500	1047.69	121.92	1723.95	265.67
	1000	1898.07	184.05	2925.61	24.75
**APG**	100	650.72	110.76	821.50	36.89
	500	1535.23	36.55	2058.91	57.26
	1000	1885.12	345.17	2886.24	38.08

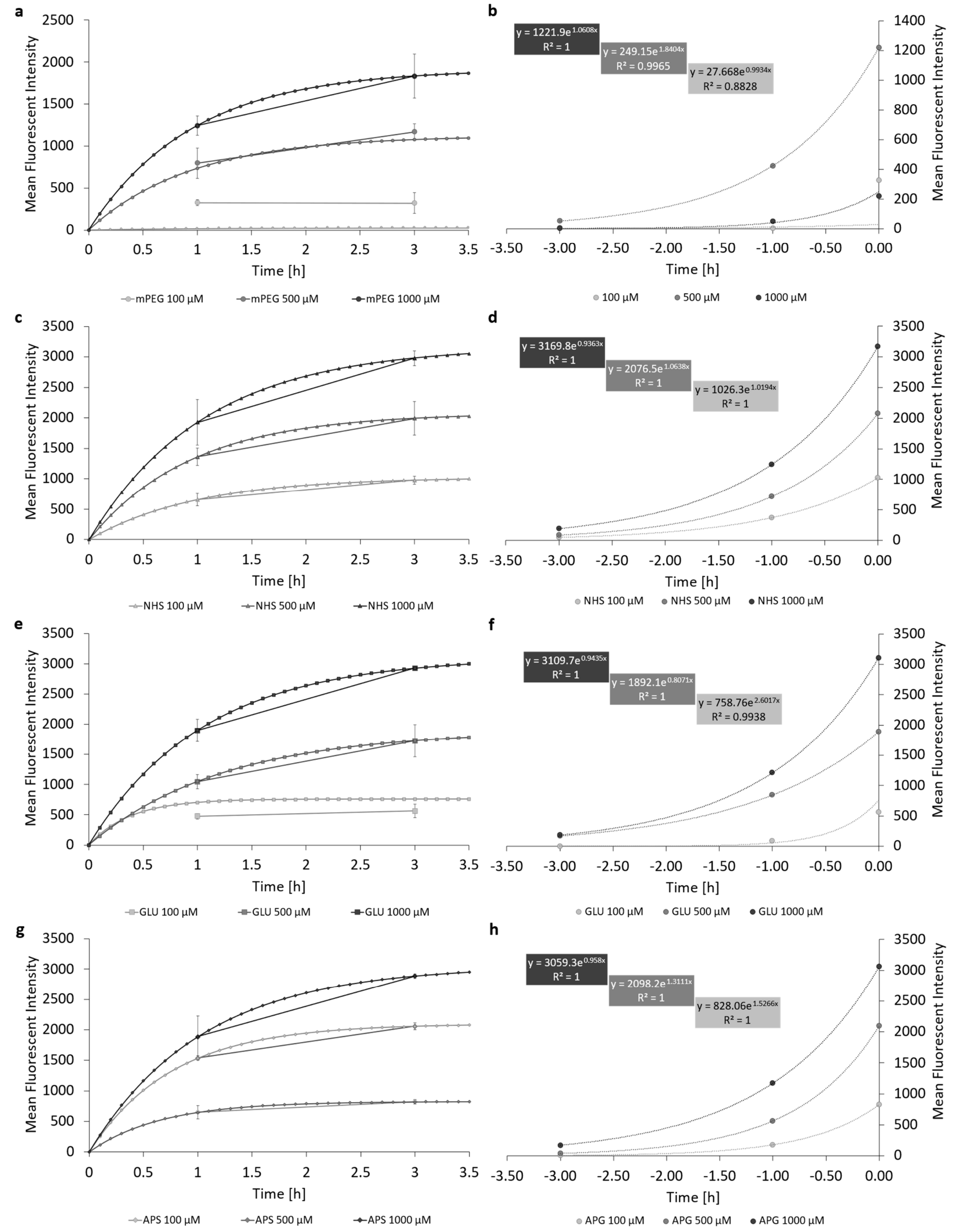

Supplementary Figure S5. Plot of the mean fluorescence intensities over time (a, c, e and g) and the corresponding exponential fits (b, d, f and h) for the cellular uptake of the different liposomal formulations into the U-87 MG cells, where. (a and b) represent the data for the mPEG liposomes, (c and d) for the NHS liposomes, (e and f) for the GLU liposomes and (g and h) for the APG liposomes.

Supplementary Table S7. Calculated cellular liposomal saturation (*S*) and the corresponding saturation half-time ($t_{1/2}$) for U-87 MG cells.

Type of liposome	Concentration [µM]	Fluorescence intensity at saturation conditions	time to reach half saturation conditions [min]
**mPEG**	100	27.668	41.9
	500	1221.9	39.2
	1000	1915.8	39.7
**NHS**	100	1026.3	40.8
	500	2076.5	39.1
	1000	3169.8	44.4
**GLU**	100	758.76	16.0
	500	1892.1	51.5
	1000	3109.7	44.1
**APG**	100	828.06	27.2
	500	2098.2	31.7
	1000	3059.3	43.4

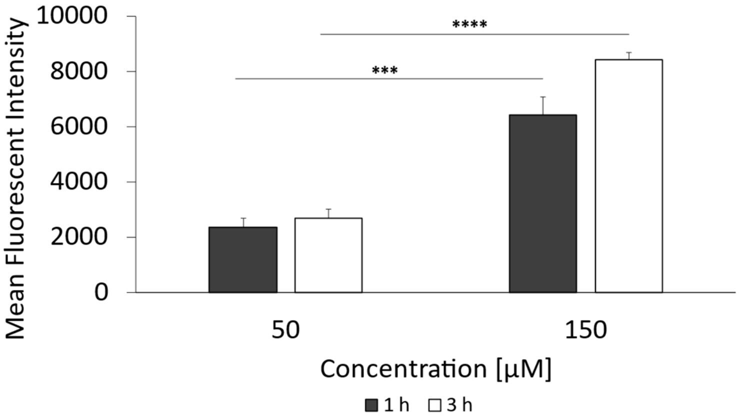

Supplementary Figure S6. Cellular uptake of 2-NBDG in U-87 MG cells for two different times of incubation (one and three hours). The bars represent the mean ± SD. Statistical analysis: one-way ANOVA followed by a two-sample t-test assuming equal variance, ***$p < 0.001$, ****$p < 0.0001$, n = 3.

Supplementary Table S8. Mean fluorescent intensities (MFI) for the uptake of 2-NBDG in U-87 MG glioma cells, n = 3.

	Concentration [µM]	1 hour of incubation		3 hours of incubation	
		Mean	SD	Mean	SD
**2-NBDG**	50	2351.50	344.68	2691.80	311.90
	150	6414.89	674.67	8420.58	263.90

Supplementary Table S9. Values of the relative viability of the bEnd.3 cells, n = 3.

Type of liposome	Concentration [µM]	Relative Viability [%]	
		Mean	SD
mPEG	100	99.2	4.1
	500	87.0	10.7
	1000	78.0	7.2
NHS	100	102.2	5.8
	500	83.7	13.6
	1000	77.1	4.4
GLU	100	87.3	4.4
	500	68.8	4.6
	1000	62.2	7.9
APG	100	99.7	5.4
	500	94.2	8.0
	1000	71.5	1.2

Supplementary Table S10. Values of the relative viability of the U-87 MG cells, n = 3.

Type of liposome	Concentration [µM]	Relative Viability [%]	
		Mean	SD
mPEG	100	99.4	5.4
	500	72.8	6.6
	1000	42.4	5.3
NHS	100	98.7	7.7
	500	74.2	10.7
	1000	58.8	2.2
GLU	100	103.5	4.4
	500	78.5	8.1
	1000	61.8	16.2
APG	100	98.3	8.1
	500	79.1	7.7
	1000	57.0	2.6
